# The dynamic impact mechanism of China's financial conditions on real economy and international crude oil market

**DOI:** 10.1016/j.heliyon.2023.e21085

**Published:** 2023-10-17

**Authors:** Jiahui Li, Hongming Li, Yuanying Jiang

**Affiliations:** a College of Science, Guilin University of Technology, Guilin, 541004, China; b Guangxi Colleges and Universities Key Laboratory of Applied Statistics, Guilin, 541004, China

**Keywords:** Financial Condition Index (FCI), Dynamic model averaging (DMA), Time-varying parameter, TVP-FAVAR, Markov chain Monte Carlo

## Abstract

As financial conditions become more complex and variable, capturing economic patterns becomes harder. The Financial Conditions Index (FCI) has gained traction as a tool to assess the performance of financial markets in nations or regions. This paragraph has created the China FCI using various financial indicators from 2002 to 2022. And with the use of statistical models like DMA-TVP-FAVAR, mixed-frequency Granger causality test, TVP-SV-VAR, and MS-VAR to analyze the relationship between China's financial condition, real economy, and the crude oil market. Different impacts were observed over time and in response to economic shocks, Results show that the fluctuation of international oil price has a negative impact on our financial condition. Therefore, the government should consider the impact of external shock factors such as international crude oil price when formulating financial policies to prevent financial risks.

## 1 Introduction

In 2022, multiple crises such as global climate change, the Russian-Ukrainian military conflict, the COVID-19 epidemic, and economic recession will bring uncertainty to global economic development. Due to global economic and financial integration, economic and financial development are increasingly linked, while the external economic situation is intricate, China's financial market is facing many risks. In order to actively and effectively respond to changes in the domestic and international situation also reduce external shocks impact on China's financial market, we should expedite the development of a new model with international circulation as the mainstay, as well as domestic and international circulation promoting each other; but also improve the modernization of financial supervision, strengthen the financial stability protection system, put all financial activities under legal supervision and ensure that no systemic financial risks. Financial development needs to serve the real economy. Therefore, studying the operation of China's financial market is an important guide to accurately understand future macroeconomic development.

The first indicator of financial market conditions was proposed by the Bank of Canada in 1994 as an index of monetary policy operating objectives, later called the Monetary Conditions Index (MCI). Therefore, Goodhart and Hofmann [1] proposed the Financial Conditions Index (FCI), which is based on MCI and includes asset price indicators such as stock prices and house prices, is widely adopted because it can reflect the financial market performance in a comprehensive way. This shows that creation of the China FCI is an important guide to scientifically control the development of the financial market and scientifically formulate monetary policy. In recent years, more and more countries are using the FCI to measure the operation of their financial markets. In the context of China's continuing reform and opening-up, financial market structure is becoming increasingly comprehensive; therefore, it is necessary to construct the FCI from a variety of perspectives to analyze and monitor China's financial market. For the central bank to precisely regulate the growth of it, the scientific and efficient development of the FCI is therefore of both theoretical and practical significance.

From the theoretical standpoint, variables of FCI constructed by the existing literature are mostly subjectively set and relatively fixed, without considering the time-varying structure of financial system. Variables which had no influence on the financial market condition in the past will increase in strength with the reform of the system and the change of time; while the variables that had a greater influence on the financial market in the past are weakened by the structural changes of the financial system, and at this time, they are still included in the indicator system would become meaningless. However, TVP-FAVAR model based on DMA, allows the weights of financial market fundamentals and coefficient weights to change dynamically over time, can well compensate for this shortcoming and improve the accuracy of research results.

From the practical standpoint, continued effect of the COVID-19 epidemic in 2022 and the double affect of the Russia-Ukraine conflict disrupt the equilibrium of crude oil market, and the emergence of unstable factors led to the skyrocketing price of crude oil. As a large importer of crude oil, China's dependence on crude oil is increasing. As a result, the operation of real economy and financial market are highly sensitive to the changes of international oil prices. In February 2022, the price of the petrochemical sector of the Chinese stock market began to fluctuate greatly. The development of China's real economy is adversely affected by the rise in automobile transportation costs caused by the skyrocketing oil prices. Therefore, it is of great practical significance to study the relationship between the international oil price and the real economy and financial conditions in China for promoting the high-quality development of economy and the stability of financial situation. With analyze the dynamic impact of China's financial condition on the real economy and the international crude oil market, which is conducive to a profound analysis of the mechanism of action of the changes of China's financial market on the real economy and provides empirical evidence for the government to achieve the goal of “steady growth” of the macro economy.

The remaining parts of this paper are structured as follows: Section 2 performs a review of the pertinent studies with point out the shortcomings of existing literature. The model principles and empirical models are laid out with Section 3. In Section 4, DMA-TVP-FAVAR is used to select a series of variables to determine China FCI. Section 5 is a study of dynamic impact mechanism among China's financial conditions, real economy, and crude oil market. To address the time-varying and stage-specific nature of the impact relationship among these three, also uses TVP-SV-VAR and MS-VAR model to conduct the empirical analysis. Finally, the conclusions are summarized, followed by policy recommendations for the relevant departments in the context of the actual situation in China.

The DMA method is applied to TVP-FAVAR model to construct DMA-TVP-FAVAR model, which considers time-varying nature of the weights and dynamic change features of the indicator system over time while measuring China's financial conditions; (2) To test the validity of the constructed China FCI, the MF-VAR model is used to explore Granger causality between the monthly FCI and quarterly GDP growth rate. The results indicate that the China FCI can be used as a short-term predictor of economic growth. (3) To address possible time-varying and stage-dependent effects between the China's financial condition, real economy and international crude oil market, this paper employs TVP-SV-VAR and MS-VAR model to examine the time-varying impulse responses even the zoned impulse responses among the three. In contrast to linear model, nonlinear model can analyze the dynamic mechanism between China's financial condition, real economy, and international crude oil market at different stages of time in a more detailed and accurate way.

## 2 Literature review

### 2.1 Financial conditions and international oil market

Regarding the connection with financial conditions and international oil prices, most studies have focused on a single market and international oil prices. And the existing literature suggests the fluctuation of international crude oil prices has a substantial effect on stock market returns, for example, Sim and Zhou [2], Eraslan and Ali [3], Wang et al. [4], Cheema and Scrimgeour [5] have demonstrated its impact. Cunado and de Gracia [6] used Vector Autoregressive (VAR) and Vector Error Correction Models (VECM) to examine the impact of oil price shocks on stock returns in 12 oil importing European economies concluded that changes in oil prices had a significant negative effect on the returns of most European stock markets. Moreover, they found that stock market was driven primarily by oil supply shocks. Ji et al. [7] and Yu et al. [8] used GARCH models to find crude oil and stock markets become more dependent around the 2008 financial crisis, and there is a tail-dependence asymmetry with significant volatility spillover.

In addition to the linkage with the stock market, other financial markets also have complex links with crude oil prices. According to Chen et al. [9], impact of oil on exchange rates became more pronounced after the financial crisis. Liu et al. [10] selected three types of commodity futures return data, including Shanghai copper, Shanghai rubber and soybean, and analyzed them based on correlation structure breakpoints and VaR quantile regression models. Nong et al. [11] found that the dynamic link between crude oil and renewable energy companies' stock prices was found to be more significant and dynamically correlated due to the frequency of financial market events. Ren et al. [12] decompose oil shocks according to oil price movements and reveal that various oil shocks have distinct dynamic effects on stock price movements. Ma et al. [13] found that if access, depth and efficiency of financial institutions and markets in developing countries could be improved, then this would significantly reduce their energy consumption intensity. Vine-Copula-CoVaR and multivariate GARCH models were used by Zeng et al. [14] to get the empirical conclusion: It is more probable that risk will be transmitted from the WTI crude oil futures market to the Chinese financial market.

Existing literature with connection about international oil prices and financial sector is gradually diversifying and comprehensive, but there is less discussion on the mechanism of the influence of Chinese financial markets on international oil prices. As the opening process of China's financial market continues to advance, is there any significant change in an effect of the gradual opening of financial market on international oil prices? In addition, whether there is asymmetry and important time-variation. In the influence mechanism that links China's financial market to international oil prices is also an important concern of this paper's empirical study.

### 2.2 Financial conditions and real economy

While finance “serves” the real economy, it also “manages” how resources are distributed in real economy. From a service perspective, the real sector dominates, and financial activities must support the growth of real economy. At same time, however, finance may also dominate the interaction across economy and financial sector: the financial sector itself, as an important tool for allocating resources in time and space, may perform “steering” position for expanding real economy. Generally, relationship between finance and the real economy is mutually reinforcing. Patrick [15], a representative study, pointed out that there may be a causal and primary connection in economic development and financial progress, with explained two different financial paths of “supply-oriented” and “demand-following”. Lack [16], Khundrakpam et al. [17], Kapetanios et al. [18] show that their constructed FCI has some predictive power for GDP and a more accurate positive effect on inflation. Taveeapiradeecharoen et al. [19] proposed a random compression method using 30 foreign exchange data (forex) pairs to reduce a large dimension of data into a smaller matrix. Then the Bayesian model average (BMA) method is used to weight the weights of each random compressed VAR to obtain the best prediction model. The results show that predicting EUR-TRY forex datasets may obtain the greatest economic benefit. Fry [20], Rousseau and Wachtel [21], Jokipii and Monnin [22] argue that the real economy has always benefited from financial development, and this benefit becomes more pronounced as degree of financial development increases. To study the time-varying impact of the U.S. financial condition index (UFCI) on inflation in China, Feng et al. [23] employ a TVP-VAR model which demonstrate that the UFCI affects inflation mainly through the Chinese financial market. Matei [24] finds that when a linear relationship is imposed, financial progress only has a short-term favorable impact on economic growth. When nonlinear hypothesis is imposed, the relationship is inverted U-shaped, because financial development positively affects economic activity before turning negative after a certain point, revealing that we need to pay more attention to the question of the pathways and mechanisms by which financial development acts on economic growth.

To further explore the above studies, this paper incorporates financial market conditions, real economic growth, and international crude oil prices into a dynamic time-varying framework, thus providing a new perspective on the interdependence between the Chinese economy, financial markets, and international energy markets.

### 2.3 International oil market and real economy

The 1970s oil crisis brought attention to macroeconomic impact of oil prices. Hamilton [25], who pioneered this field, showed that the sharp fluctuations in international crude oil prices from 1948 to 1972 were an important factor in the U.S. economic depression. In addition to studying whether international oil shocks affect the macro economy, the transmission path of changes in oil price to it has also been widely discussed, Kilian [26] and Segal [27] summarize a transmission mechanism for oil prices to macroeconomics to be the supply channel and the demand channel. The supply channel argues that rising cost of oil will have a direct impact on real economy through production, as the total social supply will decrease and potential output will fall due to increased production costs and lower output, which may eventually cause economic problems such as structural unemployment after a series of chain reactions, with representative articles including Finn [28] and Herrera [29]. The demand channel argues that residents may curtail their purchases of energy-consuming consumer durables due to higher oil prices, causing lower levels of social consumption.

Regarding the impact mechanism of international oil prices and real economy, some literature starts with an implication of crude oil in economic growth and aggregates. Caballero et al. [30] argue that the reallocation of capital among commodities, oil, and the US dollar determines the external stability of the US economy in aftermath of the financial crisis. Kilian [31] decomposes oil price shocks using a SVAR model to examine how the volatility of crude oil affects economic growth. Gómez-Loscos et al. [32], using G7 countries find that the impact of global crude oil price on economic is no longer as significant as it was in the 1970s. Alkhathlan [33] shows that domestic oil consumption of the industrial sector in Saudi Arabia has a detrimental short and long-term impact on GDP. The Russia's economy is extremely sensitive to considerable changes in oil prices, according to Benedictow et al. [34], although there is little indication of significant economic growth in the presence of higher oil prices. The effects of oil price shocks on developing net oil-importing nations are examined by Gershon et al. [35] and conclude that higher oil prices increase the per capita GDP of the selected countries in the short term.

Reviewing the literature, we find that more studies concentrate on the effects of changes in global oil prices on the real economy rather than the effects of changes in the real economy on oil prices, with fewer scholars would include studies on the short and long-term as well as nonlinear (asymmetric) relationships between international oil price changes and the real economy in the same research framework.

There is room for improvement in the existing studies as follows: First, in practice, most literatures provide abundant theoretical and empirical evidence for the pairings between financial conditions, real economy and international oil prices, but rarely analyze the three under the same framework, lacking empirical evidence of the correlation among the three. Therefore, this paper will analyze the potential relationship between the three models through the TVP-SV-VAR model and MS-VAR model. Second, the modeling ideas need to be improved, and most of the literature directly carries out empirical analysis under the set model, which lacks model persuasion. In addition, the research shows that the financial market and international oil prices, China's financial and economic development, international oil prices and economic growth have obvious nonlinear relations. If the theoretical model is assumed to be linear or the empirical research is carried out under the framework of constant parameters, the model is not persuasive. Therefore, in this paper, DMA method and TVP-FAVAR model are combined to construct the index of China's financial conditions, considering the time-varying weight and the dynamic change characteristics of the index system over time. At the same time, the time-varying impulse response function is constructed to analyze the time-varying dynamic relationship between international crude oil prices, China's financial conditions and economic development, and to capture the time-varying characteristics, random fluctuations, and transmission effects of the three, providing a new perspective for the reform of China's financial system and the healthy development of financial markets.

## 3 Materials and methods

### 3.1 TVP-FAVAR model

The basic setting equation of order TVP-FAVAR is as follows:

(1) $$x_{t}^{\left(j\right)}=\lambda_{t}^{y\left(j\right)}y_{t}+\lambda_{t}^{f\left(j\right)}f_{t}^{\left(j\right)}+u_{t}^{\left(j\right)}$$

(2) $$\left[\begin{array}{l} y_{t}\\ f_{t}^{\left(j\right)} \end{array}\right]=c_{t}^{\left(j\right)}+B_{t,1}^{\left(j\right)}\left[\begin{array}{l} y_{t-1}\\ f_{t-1}^{\left(j\right)} \end{array}\right]+\cdots +B_{t,p}^{\left(j\right)}\left[\begin{array}{l} y_{t-p}\\ f_{t-p}^{\left(j\right)} \end{array}\right]+\varepsilon_{t}^{\left(j\right)}$$

$x_{t}$ as an n-dimensional row vector containing all financial variables used to construct the FCI, $y_{t}$ as macroeconomic variables, including output and inflation rate. $\lambda_{t}^{y}$, $\lambda_{t}^{f}$ and $f_{t}$ are regression coefficients, factor loadings and co-factors, respectively. $B_{t,p}$ is the coefficient of VAR model, and $\mu_{t}$ and $\varepsilon_{t}$ are residual.

Set $\lambda_{t}={\left({\left(\lambda_{t}^{y}\right)}^{\prime },{\left(\lambda_{t}^{f}\right)}^{\prime }\right)}^{\prime }$, $\beta_{t}={\left({c_{t}}^{\prime },vec{\left(B_{t,1}\right)}^{\prime },\cdots ,vec{\left(B_{t,p}\right)}^{\prime }⥂\right)}^{\prime }$ both subject to the random walk process: $\lambda_{t}=\lambda_{t-1}+v_{t}$, $\beta_{t}=\beta_{t-1}+\eta_{t}$.

TVP-FAVAR model combines the advantages of time-varying parameters and factor augmenting vector autoregressive model, makes up for the disadvantages of traditional VAR limited by the number of variables, and can accommodate a large amount of information for factor extraction.

### 3.2 DMA method

DMA method is an extension of TVP model. The coefficient of the set explanatory variable in TVP model is time-varying, which is more advanced than the AR model with fixed parameters. However, the TVP model still sets that the variable Settings are fixed in different periods, but the contribution of the same financial variable to the financial condition index is different in different periods. After market reform and changes in the international financial environment, the financial variable that has contributed a lot to the financial condition index in the past may have a smaller influence on the financial condition index in the future.

DMA not only sets the regression coefficient to have time variability, but also allows the variable settings at different times to have time variability. The model is set as follows: $y_{t}=x_{t-1}^{\left(k\right)}\beta_{t}^{\left(k\right)}+\varepsilon_{t}^{\left(k\right)}$, $\beta_{t}^{\left(k\right)}=\beta_{t-1}^{\left(k\right)}+\eta_{t}^{\left(k\right)}$.

Among them k=1,2,⋯,K, the DMA method considers the possibility of each financial variable entering or not entering the model, so that $K=2^{m}$ combinations of variable settings can be built, so as to carry out $K=2^{m}$ times modeling and make up for the fixed defect of variable Settings.

The DMA method calculates the probability of each model containing specific financial variables at each time point, and then uses the probability to weighted average the financial condition index calculated by all models to get the final financial condition index. It uses Kalman filter method to estimate, which can reduce the operational complexity, but the accuracy will not decrease significantly. This method introduces the covariance matrix $\beta_{t-1}^{\left(k\right)}$ simplified by the forgetting factor λ, which $\lambda^{j}$ represents the proportion of the effect of the observed value of the *j* period on the current period. The simplified equation is as follows:

(3) $$\beta_{t|t-1}^{\left(k\right)}=\beta_{t-1|t-1}^{\left(k\right)}$$

(4) $$\sum_{t|t-1}^{\left(k\right)}=\frac{1}{\lambda }\sum_{t-1|t-1}^{\left(k\right)}$$

Where $\sum_{t|t-1}^{\left(k\right)}$ is the covariance matrix of $\beta_{t-1|t-1}^{\left(k\right)}$. DMA calculates the probability of all models at every moment and uses the probability as a weight to get the final index, and comprehensively considers the possibility of each variable entering or not entering the model. Compared with the traditional model, the information increment is larger, and the variable setting has time variability, which has great advantages in practice.

### 3.3 DMA-TVP-FAVAR model

The DMA-TVP-FAVAR combines DMA process and the TVP-FAVAR, drawing on advantages of both, also introduces a large number of variables into the vector autoregressive model for extracting the FCI by using the factor augmentation technique and the DMA method is used to make the variable settings time-varying at every single point of time, so that the final calculated FCI is more consistent with the reality. The basic settings of DMA-TVP-FAVAR are outlined below:

(5) $$x_{t}^{\left(j\right)}=\lambda_{t}^{y\left(j\right)}y_{t}+\lambda_{t}^{f\left(j\right)}f_{t}^{\left(j\right)}+u_{t}^{\left(j\right)}$$

(6) $$\left[\begin{array}{l} y_{t}\\ f_{t}^{\left(j\right)} \end{array}\right]=c_{t}^{\left(j\right)}+B_{t,1}^{\left(j\right)}\left[\begin{array}{l} y_{t-1}\\ f_{t-1}^{\left(j\right)} \end{array}\right]+\cdots +B_{t,p}^{\left(j\right)}\left[\begin{array}{l} y_{t-p}\\ f_{t-p}^{\left(j\right)} \end{array}\right]+\varepsilon_{t}^{\left(j\right)}$$

where $x_{t}^{\left(j\right)}$ is a row vector of several variables in the indicator pool $x_{t}$ , which constitutes the set of variables entering model *j*. $f_{t}^{\left(j\right)}$ is the index of financial condition calculated by model *j*. If *n* variables are set to always enter the model, the number of models becomes $K=2^{m+n}$. Eqs (5), (6) have the same concept as Eqs (1), (2). The corner labels (*j*) on each variable and parameter represent the equation of specific model *j*. Then the probability of each model at every moment is computed by setting the initial value $\pi_{0|0,j}$. Raftery et al. [36] create a model prediction equation with a forgetting factor α:

(7) $$\pi_{t|t-1,j}=\frac{\pi_{t-1|t-1,j}^{\alpha }}{\sum_{l=1}^{J}\pi_{t-1|t-1,l}^{\alpha }}$$

where $\pi_{t-1|t-1,j}$ is posterior probability at the t−1 time, and the update equation for the posterior probability is:

(8) $$\pi_{t|t,j}=\frac{\pi_{t|t-1,j}f_{j}\left(Data_{t}|Data_{1:t-1}\right)}{\sum_{l=1}^{J}\pi_{t|t-1,l}f_{l}\left(Data_{t}|Data_{1:t-1}\right)}$$

where $f_{j}\left(Data_{t}|Data_{1:t-1}\right)$ denotes the probability density of model *j* up to moment *t-1*, and Eq (8) gives the iterative equation for the posterior probability, thus ensuring that the model can be run all the time.

### 3.4 TVP-SV-VAR model

Since Sims [37] first suggested the VAR model, it has been extensively employed to various statistical modeling and macroeconomic forecasting applications. Given that some sample series are time-varying, time-varying elements are also included in VAR models. As research progressed and developed, Primiceri [38] was the first to introduce a TVP-SV-VAR with time-varying and stochastic volatility characteristics to analyze U.S. monetary policy and private component of national economy, this modeling framework could more adequately capture the structural variability characteristics of the U.S. economy, according to the empirical research. Since then, the TVP-SV-VAR has been widely used in the field of macroeconomics.

The TVP-SV-VAR is obtained by deriving traditional VAR; the traditional VAR has following form:

(9) $$Ay_{t}=F_{1}y_{t-1}+F_{2}y_{t-2}+\ldots +F_{s}y_{t-s}+\mu_{t}，t=s+1,s+2,\ldots ,n$$

in Eq (9), $y_{t}$ is a k×1 vector of observed variables, *t* is the time, *s* refers to the lag times, $A,F_{1},F_{2},\cdots ,F_{s}$ are all k×k matrices of coefficients, $\mu_{t}$ is used to measure structural effects, $\mu_{t}∼N\left(0,\sum \sum \right)$.

(10) $$\sum =\left(\begin{array}{cccc} \sigma_{1} & 0 & \cdots & 0\\ 0 & \ddots & \ddots & 0\\ \vdots & \ddots & \ddots & 0\\ 0 & \cdots & 0 & \sigma_{k} \end{array}\right)$$

Then we assume A is a lower triangle matrix.

(11) $$A=\left(\begin{array}{cccc} 1 & 0 & \cdots & 0\\ a_{21} & \ddots & \ddots & \vdots \\ \vdots & \ddots & \ddots & 0\\ a_{k1} & \cdots & a_{k,k-1} & 1 \end{array}\right)$$

Model (11) can be transformed into the following VAR model by simplification:

(12) $$y_{t}=B_{1}y_{t-1}+B_{2}y_{t-2}+\ldots +B_{s}y_{t-s}+A^{-1}\sum \varepsilon_{t},\varepsilon_{t}∼N\left(0,I_{k}\right)$$

where $B_{i}=A^{-1}F_{i}$, i=1,2,…,s , superimpose the elements in the row $B_{i}$ to form *β*, *β* is the vector of $k^{2}s\times 1$ , $X_{t}=I_{k}⊗\left(y_{t-1},y_{t-2},\ldots ,y_{t-s}\right)$ where ⊗ denotes the Kronecker product and model (12) can be rewritten as:

(13) $$y_{t}=X_{t}\beta +A^{-1}\sum \varepsilon_{t}$$

At this point all parameters in model (13) are fixed and do not have time-variability. Assuming that model parameters have time-variability and have SV characteristics, we can extend traditional VAR into a TVP-SV-VAR by introducing time-variability and SV characteristics, and here is the extended TVP-SV-VAR:

(14) $$y_{t}=X_{t}\beta_{t}+A_{t}^{-1}\sum_{t}\varepsilon_{t}，t=s+1,s+2,\ldots ,n$$

from this, coefficients $\beta_{t}$, parameters $A_{t}$ and $\sum_{t}$ have time-varying characteristics. Let $a_{t}=\left(a_{21},a_{31},a_{32},a_{41},\ldots ,a_{k},a_{k-1}\right)$ be the stacked vector of lower triangular elements in $A_{t}$ where $\sigma_{jt}^{2}=\exp\left(h_{jt}\right)$, the stochastic volatility matrix $h_{t}=\left(h_{1t},h_{2t},\ldots ,h_{kt}\right),j=1,2,\ldots ,k,t=s+1,s+2,\ldots ,n$. Assume that parameters in model (14) obey random walk process:

(15) $$\beta_{t+1}=\beta_{t}+\mu_{\beta t},a_{t+1}=a_{t}+\mu_{at},h_{t+1}=h_{t}+\mu_{ht}$$

(16) $$\left(\begin{array}{c} \varepsilon_{t}\\ \mu_{\beta t}\\ \mu_{at}\\ \mu_{ht} \end{array}\right)∼N\left(0,\left(\begin{array}{cccc} I & O & O & O\\ O & \sum_{\beta } & O & O\\ O & O & \sum_{a} & O\\ O & O & O & \sum_{h} \end{array}\right)\right)$$

where t=s+1,s+2,…,n, $\beta_{s+1}∼N\left(\mu_{\beta 0},\sum_{\beta 0}\right)$, $a_{s+1}∼N\left(\mu_{a0},\sum_{a0}\right)$, and $h_{s+1}∼N\left(\mu_{h0},\sum_{h0}\right)$. The model has the following assumptions, assuming that the parameters all obey first-order random walk and that random shocks to time-varying parameters are uncorrelated with each other, and that $\sum_{\beta }$, $\sum_{a}$, $\sum_{h}$ are diagonal matrices to simplify the estimation of model.

An inclusion of time-varying parameters has a significant effect on the flexibility of the model inscription, but the nonlinear state equations included in the model make the likelihood function more complicated, and it is difficult to use the great likelihood estimation. To solve this problem, the MCMC algorithm is used instead of the great likelihood estimation. In this paper, the MCMC algorithm proposed by Nakajima [39] is used for estimation, which operates in the context of Bayesian theory to obtain the joint posterior distribution of the parameters of interest. With given data, stable distribution after limit distribution is made to be the desired posterior distribution by simulating the Markov chain by repeating multiple sampling based on the previously set prior probability density. After years of development, there are many methods to construct Markov chains, Gibbs sampling is one of them, and this paper uses the MCMC method under Gibbs sampling. Next we assume that the observed sample data is known, $y={\left\{y_{t}\right\}}_{t-1}^{n}$ , $\omega =\left(\sum_{\beta },\sum_{a},\sum_{h}\right)$, and let prior density of unknown parameters ω be π(ω) , also to obtain joint posterior distribution π(ω,a,h|y) , we draw samples from the posterior distribution π(β,a,h,ω|y), and MCMC algorithm used is based on the block sampling strategy of Nakajima [39].

### 3.5 MS-VAR model

MS-VAR models are suitable for analyzing the interactions between variables in different states and automatically identifying different mechanisms according to the states of the variables. In this work, combining Markov switching and VAR model，a nonlinear MS-VAR model is used to analyze the China FCI, real economy Y and international crude oil price WTI. MS-VAR models can be divided into two main categories, namely mean-jump (MSM-VAR) and intercept-dependent (MSI-VAR), which are based on the systematic rate of change of the model's mean with the zonal system. The MSM-VAR model shows that the mean changes instantaneously with the zonal system, as shown in Eq (17):

(17) $$y_{t}-\mu \left(s_{t}\right)=\beta_{1}\left(s_{t}\right)\left(y_{t-1}-\mu \left(s_{t-1}\right)\right)+\cdots +\beta_{p}\left(s_{t}\right)\left(y_{t-p}-\mu \left(s_{t-p}\right)\right)+e_{t}$$

where $s_{t}\in \left\{1,2,\ldots ,M\right\}$, $e_{t}∼NID\left(0,\sum \left(s_{t}\right)\right)$, $e_{t}$ and $s_{t}$ represent the residuals and the zone-based parameters respectively, p is the lag order, $\beta_{i}\left(s_{t}\right)$ is the zone-dependent parameters to be estimated, $y_{t}={\left(FCI,Y,WTI\right)}^{T}$, the probability of zone-shifting can be expressed as: $P_{ij}=\mathrm{Pr}\left\{s_{t}=j|s_{t-1}=i\right\},\forall i,j\in \left\{1,2,\ldots ,M\right\}$.

MSI-VAR model represents the slow change in the mean value with the change in the zone system as: $y_{t}=\alpha \left(s_{t}\right)+\beta_{1}\left(s_{t}\right)y_{t-1}+\beta_{2}\left(s_{t}\right)y_{t-2}+\ldots +\beta_{p}\left(s_{t}\right)y_{t-p}+e_{t}$.

Considering factors such as heteroskedasticity and whether the coefficients of the estimated equation are variable, the above two major categories of models can be further subdivided, and classification results are shown in Table 1.

**Table 1:** MS-VAR model classification.

	∑ Unchanged	∑ Changed
Mean	MSM-VAR	MSMH-VAR
Intercept	MSI-VAR	MSIH-VAR

## 4 Construction of China FCI

### 4.1 Variable selection and data description

In Section 4, 12 financial variables and 2 economic variables are selected in indicator pools as follows (see Table 2):

**Table 2:** System of indicators.

Variables classification	Name of variables	Abbreviations
Financial variables	Money supply M2	M2
	7-day bank pledged repo rate	BPRR
	7-day inter-bank lending rate	IBLR
	Shanghai composite index	SCI
	Investor confidence index	ICI
	National housing climate index	NHCI
	1-year deposit-loan spread	DLS
	Social financing scale	SFS
	Financial institution loan balance	FILB
	China's bond spread	CBS
	China-US bond spread	CUS
	RMB real effective exchange rate	REER
Economic variables	Growth rate of industrial value added above scale	GROF
	CPI year-on-year growth rate	CPIG

#### 4.1.1 Currency market

Money supply M2 and interest rate indicators. Money supply, as a quantitative monetary policy tool, is a leading indicator of economic development. An appropriately accommodative money supply grows at a rate that meets the needs of economic development implies good financial conditions. At the same time, interest rates, a price-based monetary policy instrument, are crucial to the transmission mechanism of monetary policy. Lessen interest rates would reduce cost of obtaining funds for enterprises while encourage development of real economy. In this paper, 7-day bank pledged repo rate and 7-day inter-bank lending rate are chosen as proxy variables for interest rates.

#### 4.1.2 Stock market

Shanghai composite index and Investor confidence index are selected as proxy variables. The stock market is a barometer of economic development, which can well predict the future economic trend, and the prosperity of the stock market means that capital is active, and enterprises can finance more capital at lower cost in the secondary market to provide blood for the development of enterprises, when finance can better serve the economy and the financial condition is in a good state.

#### 4.1.3 Real estate market

The proxy variable chosen is National housing climate index. The real estate market is an important pillar of China's economic development, while its prosperity or recession is related to the development of many upstream and downstream industries that are closely related to it, which has the power to move the whole body. Therefore, the National housing climate index is used to measure the development of real estate market, and increasing this index suggests real estate market is moving with a positive direction and the financial situation is becoming healthy.

#### 4.1.4 Financial institution

As intermediaries between the central bank and the real economy, financial institutions play the role of uploading and transmitting, and they have the pivotal responsibility of whether monetary policy can be efficiently delivered to real enterprises. In this paper, the 1-year deposit-loan spread, social financing scale and financial institution loan balance are selected as the proxy variables of the financial institution dimension. Higher deposit-loan spreads, larger social financing and loan size of financial institutions indicate better financial conditions.

#### 4.1.5 Bond market

The proxy variables are China's bond spread and China-US bond spread. A higher spread between 10-year and 1-year Chinese government bonds indicates a more optimistic expectation for the future economy, while a larger spread between 10-year China-US bond also indicates a better economic situation at home than abroad.

#### 4.1.6 Foreign exchange market

The RMB real effective exchange rate was chosen as a proxy variable. The appreciation of RMB exchange rate implies an increase in demand for RMB in the international market, but at the same time, it will also increase the export cost of Chinese goods, which will have a detrimental effect on the development of China's foreign trade and a tense financial situation in the short term.

#### 4.1.7 Macroeconomic variables

Since the model assigns weights to financial variables based on their influence on macroeconomic variables, the Growth rate of industrial value added above scale and the monthly CPI year-on-year growth rate are selected to be macroeconomic proxy variables.

To restore the historical evolution of China's financial condition as much as possible and to consider availability of data, January 2002 to June 2022 make up the chosen data window, and the data of some variables start later than 2002 due to the late publication (see Table 3). Therefore, this paper establishes unbalanced time series data, while the DMA-TVP-FAVAR model can be modeled for balanced or unbalanced time series. The reason for choosing January 2002 as the starting point is that since China's accession to WTO in December 2002, the wave of economic and financial globalization has made country's financial market interdependent with influenced by development of financial markets in other countries around the world. The China FCI at this stage better reflects the effect of external shocks to China's financial market while providing a more comprehensive reference for government departments to prevent and resolve financial risks.

**Table 3:** Start and end times of indicators.

Start time-End time	Name of variables
2002M1-2022M6	M2 BPRR IBLR SCI NHCI
	DLS SFS FILB CBS
	CUS REER GROF CPIG
2008M4-2022M6	ICI

We do HP filtering on all financial variables, and then calculates the gap value of each variable. The gap value of interest rate variables is the original value of interest rate minus the trend value, and the gap value of other variables is equal to the difference between original value and trend value of the variable and the ratio of the trend value. HP filtering is not performed on economic variables because the impulse response function of gap value in financial variables to original value of economic variables is calculated in this paper. The descriptive statistical results of each financial variable are presented in Table 4. According to the outcomes in Table 4, the standard deviation of 7-day inter-bank lending rate and 7-day bank pledged repo rate is the largest as the volatility of both is the largest, which also objectively reflects the strong sensitivity of interest rates to changes in the macro environment. In addition, the volatility of the social financing scale, the China-US bond spread and the China's bond spread rank in the second tier, with standard deviations of 0.4409, 0.3928 and 0.3238.

**Table 4:** Descriptive statistical results.

Variables	Mean	Median	Maximum	Minimum	Std.
SCI	−0.018269	−0.018732	0.545974	−0.450922	0.132147
IBLR	−0.024419	−0.021152	3.072438	−1.263417	0.663784
REER	0.001434	−0.002161	0.103338	−0.05705	0.028234
ICI	−0.001149	0.000918	0.25672	−0.288046	0.112577
NHCI	−0.001085	0.004219	0.040402	−0.067495	0.021048
DLS	−0.006791	−0.000316	0.058847	−0.1954	0.037706
M2	−0.000745	0.000128	0.034772	−0.064132	0.01475
SFS	−0.000289	−0.081176	1.405142	−0.841579	0.440967
FILB	−0.000728	0.000851	0.041955	−0.081445	0.0151
CBS	0.007661	−0.01571	1.201443	−0.994182	0.323839
CUS	0.002734	0.040658	0.908411	−1.095425	0.392825
BPRR	−0.018861	−0.090358	3.077695	−1.244507	0.684391

### 4.2 Stability test

Testing the stationarity of the variables is important prior to model construction. Each variable in this study is put to the test using ADF unit root test method, and the evidence is presented in Table 5.

**Table 5:** Unit root test results.

Variables	t-statistic	1 % critical value	5 % critical value	Conclusion
SCI	−3.330285**	−3.45695	−2.873142	stationary
NHCI	−4.010105***	−3.457173	−2.87324	stationary
DLS	−4.869781***	−3.45695	−2.873142	stationary
M2	−5.330417***	−3.458347	−2.873755	stationary
SFS	−3.523703***	−3.458225	−2.873701	stationary
FILB	−6.203118***	−3.458347	−2.873755	stationary
CBS	−6.074035***	−3.457061	−2.87319	stationary
CUS	−3.9927***	−3.45695	−2.873142	stationary
BPRR	−7.270307***	−3.45695	−2.873142	stationary
IBLR	−7.516176***	−3.45695	−2.873142	stationary
REER	−7.516176***	−3.45695	−2.873142	stationary
ICI	−5.896671***	−3.457061	−2.87319	stationary
GROF	−5.571059***	−3.996113	−3.428349	stationary
CPIG	−3.430151**	−3.458347	−2.873755	stationary

The findings of the table show that t-statistics of Shanghai composite index and CPI monthly year-on-year growth rate are less than critical value of 5 %, and series is stable under 5 %. The t-statistic of the remaining variables is lower than critical value of 1 % level, under which the sequence is stable and meets the requirements of subsequent modeling. Finally, the stationary sequence is standardized, and the standardized equation is:

(18) $$Z_{t}=\left\{ \begin{array}{l} \frac{x_{t}-\min\left(x_{t}\right)}{\max\left(x_{t}\right)-\min\left(x_{t}\right)},\text{postive}\;\text{indicator}\\ \frac{\max\left(x_{t}\right)-x_{t}}{\max\left(x_{t}\right)-\min\left(x_{t}\right)},\text{negative}\;\text{indicator} \end{array}\right.$$

Eq (18) is the formula for standardizing the positive and negative indicators.

### 4.3 Construction and analysis of China FCI

To take full advantage of the DMA-TVP-FAVAR model, the money supply M2 is set to always exist in the model, and the rest of the financial variables are alternative variables that may or may not enter the model. The reason for always having money supply M2 is that China's monetary policy is still dominated by quantitative instruments, and the movement of money supply M2 has an important impact on the trend of the FCI.

**Fig. 1:**
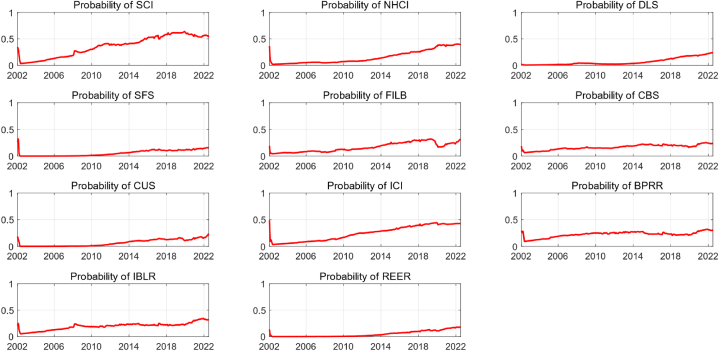
Time-varying probabilities of each financial variable entering the FCI.

Fig. 1 shows the time-varying probability of alternative financial variables entering the FCI estimated by the model. The contribution of each variable to FCI in different periods changes over time. As shown in the figure, the probability of stock price entering FCI shows an increasing trend. With the reform of market and development of economy, stock market has evolved into one of the main measures of financing enterprises and is an indelible component of Chinese financial system. The prosperity and recession of stock market have a significant influence on Chinese financial market development. Before 2008, the probability of real estate market entering FCI was relatively low. After the launch of the “4 trillion” economic stimulus plan in 2008, a large amount of capital flowed into real estate industry, which increased the real estate price. Since then, real estate industry has emerged as a significant factor influencing the growth of the economic and financial system, and the probability of entering FCI has gradually increased. However, three variables representing financial institutions, 1-year deposit-loan spread, social financing scale and financial institution loan balance, have an increasing probability of entering the FCI after 2013, and their contribution to the FCI has been increasing year by year. For bond market and currency market, the probability of entering FCI are relatively stable, without large fluctuations. And contribution of the foreign exchange market to FCI has been increasing year by year since 2015. The probability of entering FCI before 2015 is very low. This may be related to China implemented relatively fixed exchange rate system at that time. The “811″ exchange rate reform in 2015 established a managed floating exchange rate system based on market supply and demand and pegged to a basket of currencies, exchange rate is more flexible and has gradually become one of the main transmission channels of monetary policy.

**Fig. 2:**
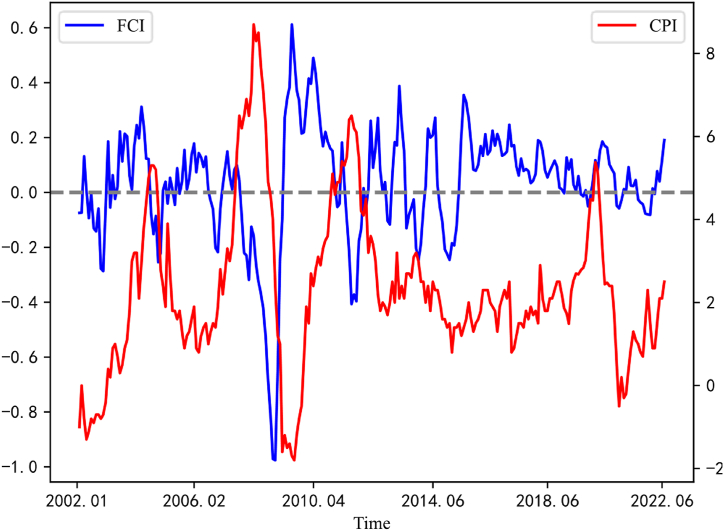
Trend of FCI and CPI.

Fig. 2 displays an FCI calculated by using DMA-TVP-FAVAR, the blue line represents FCI, while the red line represents year-over-year CPI. it can be found that during the global financial crisis in 2008 and the European debt crisis that started at the end of 2010, FCI showed a decreasing trend and was at a low level, which indicating that it has an important indication action on the occurrence of the financial crisis. Based on the association between the FCI and CPI, two basically show an inverse directional movement, indicating that the FCI can provide an effective reference for price stabilization targets in monetary policy.

The trend of the FCI during the sample period can well identify the changes in China's financial market and even macroeconomic development at different stages. The breakout of SARS in late 2002 had a short-term shock to the Chinese economy, driven by the globalization of trade, the booming foreign trade industry quickly filled the impact of the epidemic on the Chinese economy, so that after a brief decline in early 2003, the FCI began to gradually recover and even reverse its pre-epidemic level. The People's Bank of China announced the implementation of a managed floating exchange rate system on July 21, 2005. This announcement implied a change in how the exchange rate was calculated from a fixed exchange rate to a floating exchange rate and an increase in exchange rate volatility, but the exchange rate did not depreciate after the exchange reform and began to appreciate steadily without negative impact on economic development. In 2008, global financial crisis had an adverse impact on China's economy and the FCI fell sharply. To cope with the crisis, China launched a mild monetary policy to stimulate the economy and injected four trillion RMB of liquidity into the market, which improved the financial conditions at that time and the FCI began to climb steeply after a short decline. The FCI briefly declined after the European debt crisis hit China's economy at the end of 2010, followed by a sharp rise in inter-currency market lending rate in 2013, which led to a tightening of monetary liquidity and a “money shortage” and a low level of the FCI. From 2014 to 2015, the stock market saw a round of “surge - slump” and FCI also showed a V-shaped trend. In 2018, the FCI started to weaken when the trade war between the U.S. and China started and China's foreign exchange reserves decreased significantly, it adversely affected the financial market in China. 2020–2022, the domestic COVID-19 epidemic was repeated, and after each wave of epidemic peak, the Chinese government will take strong anti-epidemic measures as well as policies to promote economic recovery and contain the spread of epidemic in time, so the FCI shows a W-shaped trend.

**Fig. 3:**
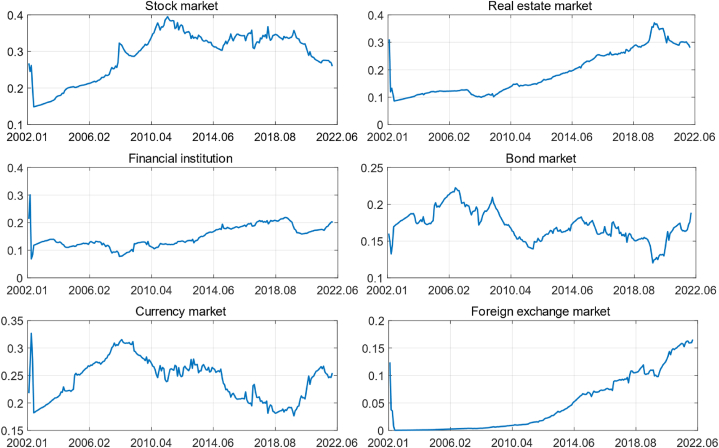
Dynamic weighting trend of each market.

The dynamic change of FCI in different periods is a comprehensive reflection about the influence change of each financial market on FCI. In this paper, the dynamic weight of each market factor is calculated according to probability of financial variables that constitute each market entering the model, and following is the formula:

(19) $$P_{i,t}=\sum_{l}p_{l,t}^{i}\frac{p_{l,t}^{i}}{\sum p_{l,t}},w_{i,t}=P_{i,t}\frac{P_{i,t}}{\sum P_{i,t}}$$

where $P_{i,t}$ is the combined probability of the financial variables constituting the market factor i after weighting, $p_{l,t}^{i}$ is the probability of the financial variables l constituting the market factor i entering the model, and $w_{i,t}$ is the weight of market factor i in t period. Weights trend of market dynamics factors is shown in Fig. 3.

From Fig. 3 we can see that the weights of the markets that constitute the FCI have been in a constant state of change, and characteristics of the dynamic weights of each market factor over the sample period can be summarized from the figure: (1) Compared with other market factors, the stock market and the currency market maintain higher weights overall. The weight of stock market showed an obvious upward trend until 2011 and remained more stable since 2011, while the weight of the currency market was in an upward trend between 2002 and 2008, then entered a slow downward trend, but changed from a downward trend to an upward trend since 2020 and the overall change was slight and stable; (2) Although the overall weights of the real estate market and foreign exchange market are not high, they have been in a rising state especially after 2008, the rising speed has accelerated, and gradually become an important factor of the FCI; (3) The weight of financial institution and bond market has a stable trend and changes more slowly.

To visually reflect the overall contribution of each market factor to the FCI, its average weight is calculated based on the time-varying weights of each market, calculated as $w_{i}=\frac{\sum_{t}^{T}w_{i,t}}{T}$.

**Fig. 4:**
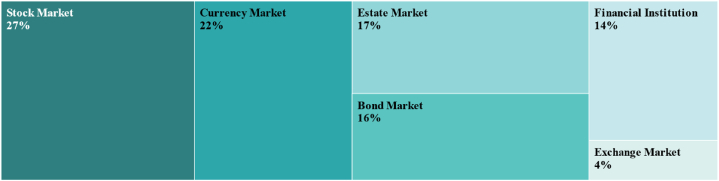
Average weighting of each market factor.

As can be seen in Fig. 4, the two factors with the largest average weights are stock market (27 %) and currency market (22 %), followed by real estate market (17 %), bond market (16 %), financial institution (14 %) and the foreign exchange market (4 %). Some conclusions can be intuitively drawn based on Fig. 4: (1) The state of China's finances is significantly influenced by the stock and currency markets. Stock market is a barometer of economic development, and the condition of economy is first reflected in stock market, while changes in currency market liquidity also have an important impact on the trend of financial conditions; (2) The average weights of the real estate market, bond market and financial institution are not very different, and their influence on financial conditions is relatively similar; (3) The average weight of the foreign exchange market on financial conditions is only 4 %, this may be related to China's managed floating exchange rate system.

### 4.4 Analysis of the correlation between FCI and economic growth in China

To test validity of the constructed China FCI, it is vital to examine the correlation between the FCI and economic growth. Existing studies mainly use Granger causality tests and construct forecasting models for analysis, mainly by summing up the FCI from monthly data to quarterly data and GDP for same frequency data matching or converting GDP from quarterly data to monthly data through quadratic interpolation, and then conducting subsequent modeling and analysis. The drawback of this approach is that when converting data of different frequencies into same-frequency data for analysis, it leads to the loss of valid information, which in turn makes the estimation results inaccurate. To solve this problem, this paper uses the mixed-frequency Granger causality test for analysis. It is based on mixed-frequency vector autoregressive model (MF-VAR), and the Wald statistic is constructed by the dimensionality reduction technique.

First of all, the mixed-frequency variables are described, $x_{H}$ as the high-frequency variable (China FCI), $x_{L}$ as the low-frequency variable (quarterly GDP growth rate) and the frequency ratio between them is set as m (the frequency ratio of quarterly GDP growth rate to China FCI is =3). In addition, $\tau_{L}$ is the time scale of low-frequency variable, and $x_{H}\left(\tau_{L},j\right)$ is the j-th high-frequency data in $\tau_{L}$ low-frequency moment. In this paper, $x_{H}\left(\tau_{L},1\right)$ is the FCI data in the first month of $\tau_{L}$ quarter. In this way, both low-frequency data and high-frequency data can be placed in a matrix of (m+1)×T dimension, assuming that the mixed-frequency data obey the MF-VAR (*p*) process, which can be expressed by the equation as

$$\left[\begin{array}{c} x_{H}\left(\tau_{L},1\right)\\ \vdots \\ x_{H}\left(\tau_{L},m\right)\\ x_{L}\left(\tau_{L}\right) \end{array}\right]=\sum_{k=1}^{P}\left[\begin{array}{cccc} d_{11,k} & \cdots & d_{1m,k} & c_{\left(k-1\right)m+1}\\ \vdots & \ddots & \vdots & \vdots \\ d_{m1,k} & \cdots & d_{mm,k} & c_{km}\\ b_{km} & \cdots & b_{\left(k-1\right)m+1} & a_{k} \end{array}\right]\left[\begin{array}{c} x_{H}\left(\tau_{L}-k,1\right)\\ \vdots \\ x_{H}\left(\tau_{L}-k,m\right)\\ x_{L}\left(\tau_{L}-k\right) \end{array}\right]+\left[\begin{array}{c} \varepsilon_{H}\left(\tau_{L},1\right)\\ \vdots \\ \varepsilon_{H}\left(\tau_{L},m\right)\\ \varepsilon_{L}\left(\tau_{L}\right) \end{array}\right]$$

It is formatted as $X\left(\tau_{L}\right)=\sum_{k=1}^{P}A_{K}X\left(\tau_{L}-k\right)+\varepsilon \left(\tau_{L}\right)$.

To test long-term causality between the mixing variables, here expands MF-VAR(*p*) to MF-VAR(*p*,*h*):

(20) $$X\left(\tau_{L}+h\right)=\sum_{k=1}^{P}A_{k}^{\left(h\right)}X\left(\tau_{L}-k+1\right)+\varepsilon^{\left(h\right)}\left(\tau_{L}\right)$$

The test of causality between the reference period of prediction and h-period mixed frequency variable requires the following constraint on (20): $H_{0}\left(h\right):Rvec\left[B\left(h\right)\right]=r$, where *B(h)* is coefficient of the MF-VAR (*p*,*h*), *R* is a row-full-rank selection matrix, and *r* is the constraint vector.

Next, this paper uses constructed MF-VAR (*p,h*) to test the Granger causality between monthly FCI and quarterly GDP growth rate. The monthly FCI is selected from the series measured above, and the quarterly GDP growth rate data is selected from the National Bureau of Statistics（NBS）. The results of the two are shown in below.

As seen by what happens in Table 6, it shows original hypothesis that FCI is a Granger cause of GDP is rejected at 10 % significance level when the reference period of prediction is h=1 , lag period is p=1,2,3. Conversely, original hypothesis that GDP is not a Granger cause of FCI is accepted at the 10 % significance level. The results of mixed-frequency Granger causality test show FCI and GDP are Granger causes of each other when the reference period of prediction is h=2 , lag period is p=1. FCI is Granger cause of GDP at 10 % significance level, but GDP is not Granger cause of FCI. When lag period is =2 , FCI is no longer the Granger cause of GDP. Therefore, the correlation between FCI and GDP is strong when the lag period is =3 , the correlation between the two is weakened or even non-existent when the lag period is greater than 2, which means that FCI can be used as a predictor of economic growth in the short term.

**Table 6:** Mixed-frequency Granger causality test of monthly FCI and quarterly GDP growth rate.

	h=1	h=2
		
p=1	$\left[\begin{array}{cc} - & 0.305\\ 0.0025 & - \end{array}\right]$	$\left[\begin{array}{cc} - & 0.0245\\ 0.01 & - \end{array}\right]$
p=2	$\left[\begin{array}{cc} - & 0.161\\ 0.002 & - \end{array}\right]$	$\left[\begin{array}{cc} - & 0.02755\\ 0.0585 & - \end{array}\right]$
p=3	$\left[\begin{array}{cc} - & 0.0525\\ 0.074 & - \end{array}\right]$	$\left[\begin{array}{cc} - & 0.069\\ 0.598 & - \end{array}\right]$

## 5 Dynamic transmission effects of financial conditions, real economy, and international oil prices

### 5.1 A first look at research mechanism

As a necessary factor of energy production in the industrial economy of various countries, oil has basic commodity attributes. Since NYMEX launched the world's first oil futures in 1978, it has developed rapidly with a variety of complex derivatives such as options. The financial nature of the world oil market has become increasingly prominent, and crude oil has gradually become an asset class. Therefore, international oil prices will trigger financial market fluctuations to a certain extent, mainly affecting the stock market, foreign exchange market and derivatives market.

The transmission of international oil price to the stock market is based on the price linkage of the global financial market and the financial characteristics of crude oil itself. First, crude oil and stocks are two products that are not completely related, with different levels of risk, and investors can choose flexibly when allocating financial assets. According to Markowitz portfolio theory, investors can adjust the proportion of financial assets to create a suitable investment portfolio to reduce investment risk and achieve the best portfolio return. Second, noise and herding increase the linkages between markets, thereby strengthening the interaction between crude oil and stock markets. In view of information asymmetry and irrational investment, when the price of a certain financial asset rises, on the one hand, investors may change their strategies according to traditional media reports, social network opinions and personal emotions, increase the holdings of this financial asset and reduce the holdings of other financial assets. On the other hand, investors also buy financial assets by observing other investors. Therefore, the combined effect of the two makes the transmission of information between markets more obvious and strengthens the spillover effect.

The transmission path of international oil price to foreign exchange market is mainly divided into two aspects: international balance of payments and price level. In the transmission process of the balance of payments, take China as an example. For countries importing crude oil, the rise in international oil prices will first increase the cost of imported crude oil. In the short term, the demand for foreign exchange will rapidly exceed the supply, resulting in the appreciation of the exchange rate and the depreciation of the local currency. In terms of price level, for countries importing crude oil, higher international oil prices will first drive the price of crude oil alternative energy, which in turn will increase the cost of other related products, and the producer price index (PPI) and consumer price index (CPI) will rise accordingly, resulting in an increase in imported inflation and a decrease in the purchasing power of domestic money leading to its depreciation.

The transmission of international oil prices to the derivatives market is mainly through two paths: spot market and price linkage. In terms of the impact on the derivatives market through the spot market, in view of the price formation and hedging function of the derivatives market, the derivatives market is inseparable from the spot market. Changes in international oil prices will cause price fluctuations in the spot market, which will lead to price changes in the derivatives market. In terms of swap shocks in derivatives markets, swaps refer to the interaction and synergy of price movements in different countries, regions, or markets. The increasing integration of crude oil and derivatives markets has accelerated the emergence of price linkages between the two markets and facilitated the transmission of shocks and information from oil price movements to derivatives market prices. As far as crude oil futures are concerned, they are important derivatives in themselves, and the price fluctuations of crude oil futures and other derivatives inevitably change the prices of these derivatives, thus affecting the derivatives market.

### 5.2 Variable selection and data description

To learn more about the dynamic mechanism of China's financial conditions, real economy, and international crude oil prices, selecting the FCI measured above, industrial added value year-on-year growth rate Y to represent the development of real economy, and WTI to characterize the trend of international crude oil prices. Industrial added value is final monetary output of enterprises' production activities during the reference period, and the development of industrial enterprises is an important part of real economy growth, and the trend of enterprise development can be a good measure of the real economy development; China's crude oil import demand has been increasing in recent years, as of July 2022, China has imported 290 million tons of crude oil cumulatively, ranking first in the world in terms of quantity, and WTI crude oil will serve as a global crude oil benchmark and can be a good measure of crude oil price fluctuations. The period chosen for the study is from January 2003 to June 2022, and Table 7 displays the descriptive statistics for the original series.

**Table 7:** Descriptive statistics results.

Variables	Mean	Median	Maximum	Minimum	Std.
FCI	0.036881	0.061507	0.612213	−0.97588	0.215327
WTI	67.65919	65.125	140.02	18.84	23.86992
Y	10.83289	9.65	52.33918	−25.86705	6.661305

As shown in Table 7, mean value of WTI is $67.66/barrel, the minimum value is only $18.84/barrel, but the maximum value reaches $140.02/barrel, indicating that the WTI is more volatile, and its maximum value reached during the financial crisis in 2008, shock of crisis led to substantial fluctuation of crude oil price. Industrial added value year-on-year growth rate Y mean value is 10.83 % with a standard deviation of 6.66, which is relatively volatile, where the maximum value is 52.33 %, which reached in February 2021, because China is in the rapid development stage of the COVID-19 epidemic in February 2020, and the economic development is restricted, so the industrial added value reaches the trough in February 2020, thus leading to Y reaches a peak in February 2021; similarly, the minimum value is −25.86 %. It is reached in February 2020 for the same reason as above.

### 5.3 Stability test and lag period selection

Time series data frequently exhibit a common time trend, and if these data are modeled directly there may be come pseudo-regression problems, so first the data must be tested for stationarity, a unit root test must be performed. The ADF test, one of the most used unit root tests, is employed in this study. Table 8 below displays the test results:

**Table 8:** Unit root test results.

Variables	t-statistic	1 % critical value	5 % critical value	Conclusion
FCI	−5.188105	−3.459898	−2.874435	stationary
WTI	−2.680866	−2.574968	−1.942199	stationary
Y	−4.021084	−3.458594	−2.873863	stationary

From the data above, it can be found that the ADF test values of FCI, crude oil price WTI and industrial added value growth Y are all below the critical value of the t-statistic at 1 % significance level, so they are all stationary data series that meet the requirements for further modeling.

Before constructing VAR model, the lag order of model should be determined first. The results in Table 9 show that the LR, FPE, AIC, and HQ parameters are considered optimal when the reference order is 2. Therefore, the lag order is set as order 2 in this paper.

**Table 9:** Lag period selection results.

Lag	LogL	LR	FPE	AIC	SC	HQ
0	−972.9066	NA	0.937795	8.449408	8.494114	8.467439
1	−432.2490	1062.591	0.009398	3.846312	4.025139*	3.918439
2	−411.8908	39.48263*	0.008518*	3.747972*	4.060919	3.874195*
3	−403.0998	16.82077	0.008534	3.749782	4.196849	3.930100

**Note:** LR: sequential modified LR test statistic; FPE: Final prediction error; AIC: Akaike information criterion; SC: Schwarz information criterion; HQ: Hannan-Quinn information criterion.

In addition, the robustness of model is tested in this paper to confirm the validity of results of the subsequent analysis. All inverse roots of the model consisting of the FCI, crude oil price WTI and year-on-year industrial added value growth Y are smaller than 1 and fall within the unit circle shows that the model is robust.

### 5.4 Model fitting results

In this study, a TVP-SV-VAR model with three variables is created, including the FCI, crude oil price WTI and year-on-year industrial added value growth rate Y. The TVP-SV-VAR is estimated using MCMC algorithm with 10,000 samples, and Table 10 provides the outcomes of the posterior distribution estimate of the model parameters. It demonstrates that the 95 % confidence interval for the mean values of the parameters that need to be estimated is within those values, and the Geweke value is below 1.96, demonstrating that the parameters follow the posterior distribution. Further evidence that the model is accurate comes from the fact that the highest value of the invalid factor is only 164.07, which is far less than 10,000.

**Table 10:** Parameter estimation of TVP-SV-VAR model.

Parameter	Mean	Stdev	95 % L	95 % U	Geweke	Inef.
sb1	0.0227	0.0026	0.0183	0.0284	0.919	12.7
sb2	0.022	0.0023	0.0179	0.027	0.228	15.3
sa1	0.0459	0.0089	0.0323	0.0671	0.447	29.18
sa2	0.0583	0.0174	0.034	0.1028	0.259	74.49
sh1	1.9536	0.21	1.5487	2.4072	0.033	164.07
sh2	0.3059	0.0797	0.1834	0.5028	0.128	73.68

### 5.5 Time-varying impulse responses analysis

The TVP-SV-VAR has its impulse response function, which better captures the phase characteristics and differences between variables at various time periods within the sampling interval compared to the linear VAR.

**Fig. 5:**
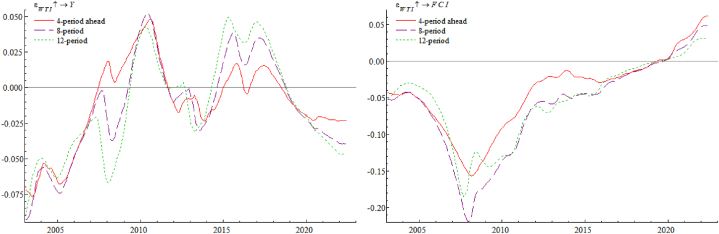
Impulse response of year-on-year industrial added value growth rate Y (Left) and FCI (Right) against international crude oil price WTI.

Fig. 5 (Left) shows the impulse response of year-on-year industrial added value growth rate Y against international crude oil price WTI. It is obvious that whether in short, medium, or long term, the rise in crude oil prices has a similar trend-like effect on real economy, and the degree of such impact will be relatively greater in long term, which suggesting that the consequences of WTI increase on real economy has a long-term nature. As appears, facing a rise WTI prices will cause enterprises' production costs to increase, their profitability and capacity utilization rates to decline, and there will be insufficient demand for investments; at the same time, and also will lead enterprises to reduce labor costs, consumers' real disposable income decreases, and effective demand is insufficient, leading to a fall in enterprise supply and a further decline in investment enthusiasm. In turn, the cost and price increases caused by sharp rise in oil prices will lead to a tightening of monetary policy. Therefore, rising international crude oil prices often represent aggregate demand in expansion, while at the end of upward cycle, when international oil prices begin to stagnate or even turn around, it often means a contraction in aggregate demand and opening of the economic downward cycle. Fig. 5 (Right) shows an impulse response of FCI against the WTI. From this figure, rise in crude oil prices mainly has a negative impact on China's FCI, with the largest lagged impact in 12 periods and the smallest lagged impact in 4 periods. For the exchange rate market, when WTI price rises, driven by the market risk aversion, the expected appreciation of the US dollar becomes a reality, which puts some pressure on the RMB exchange rate. For stock market side, as crude oil plays a productive role in factor market, its international price changes will directly lead to earnings changes by affecting costs, thus causing stock price fluctuations of listed companies. The corporate discount rate, on the other hand, consists of risk-free rate and the risk premium, and level of the risk premium is an important factor in determining discount rate of stock valuation channel. Therefore, changes in international crude oil prices can indirectly link to stock prices by affecting risk premiums, creating risks and adverse effects for the stock market. For the currency market side, as a result of rising WTI prices, it will affect production costs of enterprises, in order to maximize profits, enterprises will choose to pass on part of the costs brought by increase in crude oil prices to consumers, causing the selling price of products to rise, thus driving the expected inflation rate. At this point, the central bank, in order to reduce inflationary pressure, will implement a tight monetary policy by raising interest rates or rolling back money, driving up lending rates, raising the discount rate, and tightening money market liquidity, which ultimately has an adverse impact on financial conditions.

**Fig. 6:**
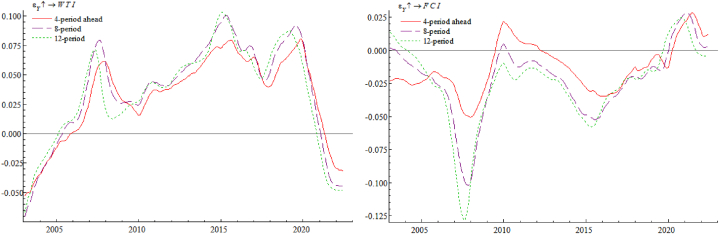
Impulse response of international crude oil price WTI (Left) and FCI (Right) against year-on-year industrial added value growth rate Y.

Fig. 6 (Left) shows an impulse response of WTI against year-on-year industrial added value growth rate Y. As can be seen from the graph, real economic growth in general drives up international oil prices. Economic growth expands aggregate demand, which leads to a further increase in aggregate supply, increasing demand for international crude oil and stimulating its prices to rise. Also from the figure, it is evident that the degree of impact of real economy on international oil prices is basically the same across lags, with a slightly larger impact in lag 12. Fig. 6 (Right) shows an impulse response of FCI against year-on-year industrial added value growth rate Y. As can be seen, the growth of real economy has a negative and then positive impact on financial conditions in short term, probably because sudden overheated growth of the economy generates a sudden increase in demand for money, and the liquidity of the financial market is unable to meet the additional demand for money caused by the sudden overheating of the economy in a short period, which in turn causes problem of tight liquidity in the currency market and financial conditions show a short-term downward trend. Subsequently, as the central bank implemented a relatively accommodative monetary policy to accommodate the overheated economic growth and replenish market liquidity in a timely manner, financial conditions gradually tended to be favorable.

**Fig. 7:**
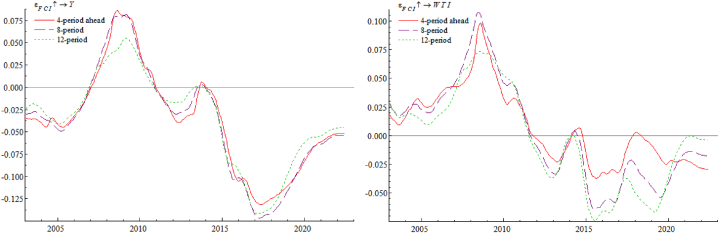
Impulse response of year-on-year industrial added value growth rate Y (Left) and international crude oil price WTI (Right) against FCI.

Fig. 7 (Left) shows the impulse response of year-on-year industrial added value growth rate Y against FCI. As can be seen, impact of better financial conditions on real economy is negative, then positive, then negative, and service effect of better financial conditions on real economy has a lagging effect, because the loose monetary policy can only be transmitted to real economy through the intermediary of commercial banks, and cannot reach the real economy directly, which leads to the lagging effect of monetary policy implementation. In addition financial conditions become better will not always benefit the real economy, if the prosperity of the financial market is largely linked to the virtual economy, resulting in a large amount of funds flowing into the virtual economy, then entrepreneurs will be less willing to invest in the real economy, a large amount of funds will eventually flow to real estate and other speculative activities, asset bubbles will become larger and larger, some funds will be stranded in the banking and financial system, buying and selling each other, transaction costs will objectively increase the financing costs, which will only squeeze the development of real economy. Fig. 7 (Right) shows an impulse response of WTI against FCI. It can be seen that better financial conditions show a positive and then negative effect on oil price. Good financial conditions inject a lot of liquidity into the financial market, and some funds will flow to international crude oil market to invest in it, thus pulling up the price. Compared with crude oil, spot precious metals market demand is not high, but crude oil is widely used as an industrial raw material in various manufacturing and refining industries and has a national strategic status, so investors have a great demand for a combination of crude oil's spot and futures hedging, and this demand will pull up the price of international crude oil.

### 5.6 Zoned impulse responses analysis

To further analyze the influence relationship between variables when economy in different states, this section uses the MS-VAR model for analysis. Before modeling with MS-VAR, the optimal lag order of model is first determined based on AIC, HQ and SC information criteria. The determination of optimal lags was done above, and this section will still follow the above results by choosing a lag of 2. Secondly, the specific form of model is set as MSIH-VAR. Table 11 presents a parameter test results for the linear VAR(2) model and the nonlinear MSIH(2)-VAR(2) model. According to the principle that the larger the log-likelihood value and the smaller the information criteria such as AIC, HQ and SC, the better the model fit is, the nonlinear MSIH(2)-VAR(2) is more realistic than linear VAR(2). In addition, the LR test score for linearity of the MSIH(2)-VAR(2) is 403.8347, and the *P*-value obtained from the Chi-square statistic is much less than 1 %, significantly rejecting the original hypothesis that the linear VAR(2) model is optimal.

**Table 11:** MSIH-VAR and VAR model parameter test results.

	MSIH(3)-VAR(2)		VAR(2)
log-likelihood	−211.8134		−413.7307
AIC	2.1536		3.7994
HQ	2.3812		3.9612
SC	2.7181		4.2005

**Fig. 8:**
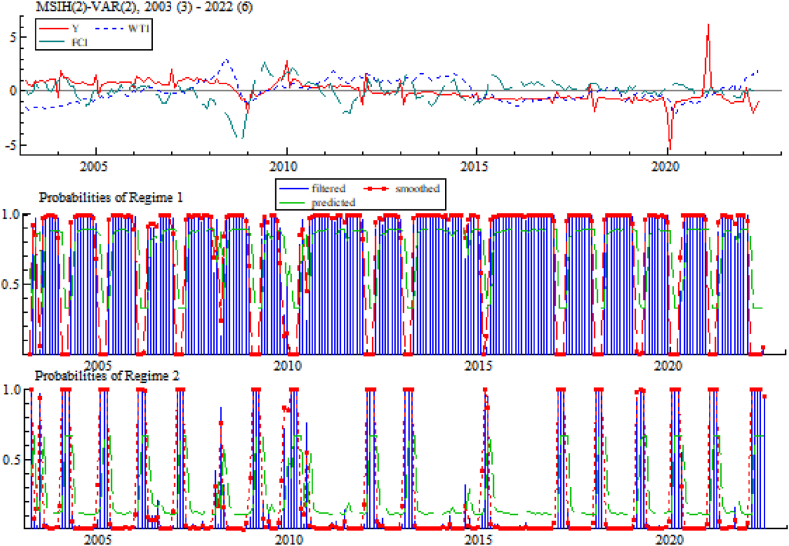
Zone system probability.

Hamilton's Expectation Maximization (EM) algorithm is used for estimation, and after a finite number of iterations, the MSIH(2)-VAR(2) model parameter estimates, sample intervals that fall within the zone system and zone system probability graph are obtained, and results are shown in Table 12, Table 13, and Fig. 8, respectively.

**Table 12:** MSIH(2)-VAR(2) model estimation results.

Variables	Y		WTI		FCI	
	coefficient	t-value	coefficient	t-value	coefficient	t-value
Intercept term （Zone system 1）	−0.018	−1.622	0.013	0.634	−0.071**	−2.264
Intercept term （Zone system 2）	0.016	0.070	0.000	0.014	0.219***	3.361
Y_1	1.074***	39.276	0.045*	1.816	−0.020	−0.468
Y_2	−0.115***	−5.336	0.002	0.089	−0.052	−1.345
WTI_1	0.008	0.171	1.122***	17.043	0.214**	2.087
WTI_2	−0.015	−0.316	−0.156**	−2.299	−0.320***	−3.038
FCI_1	−0.030	−1.282	0.091**	2.289	0.934***	15.142
FCI_2	0.059**	2.263	−0.059	−1.552	−0.117*	−1.926
Standard deviation（Zone system 1）	0.132		0.274		0.399	
Standard deviation（Zone system 2）	1.735		0.246		0.446	

**Note:** *, ** and *** indicate significant at 10 %, 5 % and 1 % confidence levels, respectively.

**Table 13:** Sample intervals that fall within the zone system.

Zone system 1	Zone system 2
2003:4–2003:5	2003:3–2003:3
2003:7–2003:12	2003:6–2003:6
2004:4–2004:12	2004:1–2004:3
2005:4–2005:12	2005:1–2005:3
2006:4–2006:12	2006:1–2006:3
2007:4–2008:2	2007:1–2007:3
2008:4–2008:12	2008:3–2008:3
2009:4–2009:10	2009:1–2009:3
2010:4–2010:5	2009:11–2010:3
2010:7–2011:12	2010:6–2010:6
2012:4–2012:12	2012:1–2012:3
2013:4–2015:1	2013:1–2013:3
2015:4–2016:12	2015:2–2015:3
2017:4–2017:12	2017:1–2017:3
2018:4–2019:1	2018:1–2018:3
2019:5–2019:12	2019:2–2019:4
2020:4–2020:12	2020:1–2020:3
2021:4–2022:1	2021:1–2021:3
–	2022:2–2022:6

Table 12 lists the parameter estimates of MSIH(2)-VAR(2) model. From volatility term where the dependent variable is year-on-year industrial added value growth rate Y, standard deviation of zone system 1 is 0.132 and the standard deviation of zone system 2 is 1.735, as the standard deviation of zone system 2 is larger and the economic volatility is stronger, the economic volatility of zone system 1 is weaker, thus naming zone system 1 as the low volatility state and zone system 2 as the high volatility state.

Table 14 summarizes the probability of transfer between zone systems, in which the probability of each zone system remaining stable is 0.8939 and 0.6723, indicating that the division of zone systems is relatively stable and reasonable; in terms of the duration of zone systems, zone system 1 (low volatility state) lasts longer compared to zone system 2 (high volatility state), and the sample size of the economy in a low volatility state accounts for 75.82 % of the total sample.

**Table 14:** Zone system transfer probability and characteristics.

	Zone system 1	Zone system 2	Sample size	Frequency	Duration
Zone system 1	0.8939	0.1061	175.1	0.7554	9.42
Zona system 2	0.3277	0.6723	56.9	0.2446	3.05

**Fig. 9:**
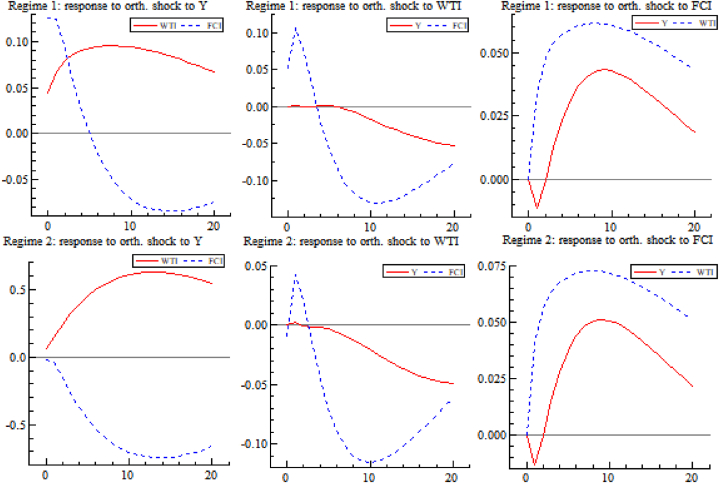
Impulse responses between variables in different zone systems.

Fig. 9 shows the impulse response between the variables for different economic states (zone systems). The first column is the response of international crude oil prices and China's financial conditions to the shocks from real economy, and shocks cause oil prices to rise regardless of the fluctuations in economy. The impact of positive economic shocks on financial condition varies, with unit economic shocks causing a positive and then negative impact on the FCI in low volatility (zone system 1) state. While in high volatility (zone system 2) state, economic growth has a negative impact on FCI. The second column shows a response of real economy and FCI by oil shocks, and the results mean that impact of international oil price increase on real economy and financial condition has similarity in different volatility states, specifically, it on financial condition shows a positive trend first and then negative trend, but it always shows negative impact on real economy. In other words, higher international oil prices will promote financial condition in short term and will play an opposite role in the long term but will always inhibit economic growth. The third column shows the response of the real economy and international oil prices by financial conditions shocks, and the results mean that in different volatility (zone system) states, positive shocks of financial conditions lead to higher international crude oil prices and mainly cause positive growth of the real economy, In other words, better financial conditions benefit the real economy.

## 6 Conclusion

The main purpose of FCI is to assess stability of financial markets and summarize volatility and interaction effects of each market segment into a continuous statistic. First, the FCI is constructed based on DMA-TVP-FAVAR and its correlation with economic growth is analyzed. In this paper, 12 financial variables are selected from the currency market, stock market, real estate market, financial institutions, bond market, and foreign exchange market, and the money supply M2 is always fixed in the model, while the remaining 11 variables are executed as dynamic model averaging and used to extract the China FCI from January 2002 to June 2022. In the next step, a mixed-frequency Granger causality test is used to analyze association with the FCI and economic growth to test the validity of the constructed China FCI so that the final calculated FCI can be more consistent with the real situation. The study's findings demonstrate that FCI can be used as a predictor of economic growth in the short term, and its trend is generally consistent with evolution of China's financial market and macroeconomic development during sample period, which is reasonable in terms of both its cyclical characteristics and its ability to identify serious financial risk events. Then the dynamic relationship between the financial conditions in China, real economy and international crude oil market is explored. The FCI, year-on-year industrial added value growth rate Y and international crude oil price indicator WTI are selected as the indicators, the monthly data with the interval from January 2003 to June 2022 are used to analyze time-varying characteristics of variables and explore dynamic links among them. Firstly, a TVP-SV-VAR model was established, and time-varying impulse response analysis was conducted. The findings indicate that: (1) Impact relationship between variables in sample interval will have phase characteristics and differential changes over time; (2) The long-term effects of international crude oil price on real economy and financial market are more significant in process of international crude oil market-China financial conditions-real economic growth interconnection. The increase in crude oil price mainly causes negative effects on China's FCI while positively affecting real economic growth. In impulse response function of China's real economy, response of international crude oil prices shows that the real economy has basically the same degree of impact on international oil prices in different lags; while for the financial market, the lagged effects show alternating positive and negative characteristics of market volatility in the current period, not a single promotion or suppression effect; (3) The impulse response function leveling effect of the financial condition shock in China has a lag, with a negative then positive then negative impact on real economy as a result of a better financial condition, while the impact on WTI has a positive then negative trend. To further analyze relationship between the variables when economy is in various states, this section also uses the MS-VAR model for analysis. By building a nonlinear MSIH(2)-VAR(2) model, the volatility of the real economy is divided into two zonal states, low volatility and high volatility, and the variability of impact between international crude oil market - China's financial conditions - real economic growth under different zonal states is investigated. The zonal impulse response analysis shows that regardless of the volatility of the real economy, its shocks lead to higher oil prices, and shocks of real economy to China's financial conditions show heterogeneous results in the two zonal regimes.

Based on the results mentioned above, following relevant aspects of policy recommendations can be obtained:

(1) Consider the FCI as one of the important “indicators” of economic policy. In the new international development context, China's financial market conditions are facing a more complex transmission environment and rapidly changing economic situation. At this time, single traditional financial intermediary variables such as interest rate and exchange rate are gradually “eclipsed” on the historical stage, but the FCI is different from them. In terms of economic early warning and realistic reflection ability, it can be regarded as one of the important indicators of economic policy.
(2) In the case of violent fluctuations in oil prices, we should resolutely avoid direct contagion and multiple shocks to China's economy and financial markets from risk of international oil price fluctuations and prevent occurrence of systemic financial risks. Seize the golden opportunity to build up strategic oil reserves as international oil prices fall. When market panic subsides and oil prices rise, oil reserves can be used to absorb the shock, thereby mitigating a series of negative shocks to stable growth of real economy and financial environment from rising oil prices.

## 7 Discussion

Limited by us, there are still some regrets in this paper, which are also the further assumptions of future research. It mainly includes the following two aspects:

(1) DMA-TVP-FAVAR is used to construct the financial condition index, which allows the parameters and index system to be time-varying, scientifically fit the actual situation to a great extent, and improve the accuracy of the research results. However, the parameter setting of DMA-TVP-FAVAR model is always time-varying, while the reform of financial system and the sudden change of financial environment are not always present in reality. Therefore, the DMA-TVP-FAVAR model with time-varying parameter setting has the defect of over-parameterization, and the evolution of parameters in the model is worthy of further improvement.
(2) In Section 5, the TVP-SV-VAR model and MS-VAR model are used to analyze the time-varying impulse response relationship and the zoning impulse response relationship between financial conditions, real economy, and financial market, which reveals the nonlinear influence relationship between financial conditions, real economy, and international crude oil market, and has certain innovation. Of course, the method selected in this section has not broken through the “dimensional curse”. In the future, the augmentation factor can be incorporated into the model to break through the “dimensional curse” and include more variables to accommodate more macro information.

## Funding statement

This project was supported by the 10.13039/501100001809National Natural Science Foundation of China (No.71963008).

## Data availability statement

Data will be made available on request.

## Additional information

No additional information is available for this paper.

## CRediT authorship contribution statement

**Jiahui Li:** Conceptualization, Methodology, Software, Validation, Writing – original draft, Writing – review & editing. **Hongming Li:** Data curation, Formal analysis. **Yuanying Jiang:** Funding acquisition, Methodology, Project administration, Resources, Supervision, Validation, Writing – review & editing.

## Declaration of competing interest

The authors declare that they have no known competing financial interests or personal relationships that could have appeared to influence the work reported in this paper.
